# Ancient Gut Microbiomes Shed Light on Modern Disease

**DOI:** 10.1289/ehp.121-a118

**Published:** 2013-04-01

**Authors:** Sharon Levy

**Affiliations:** Sharon Levy, based in Humboldt County, CA, has covered ecology, evolution, and environmental science since 1993. She is the author of *Once and Future Giants: What Ice Age Extinctions Tell Us about the Fate of Earth’s Largest Animals.*

People in westernized, urban environments host different gut microbes than people in remote, undeveloped parts of the world. Children in remote regions of Burkina Faso, for instance, have been shown to harbor bacteria alien to the guts of Europeans.^1^ A similar pattern has been found in comparing the microbiomes of rural people from Malawi and Venezuela to those of U.S. residents.^2^ Now findings from an ancient cave dwelling deserted 1,400 years ago shed new light on our shifting internal ecosystems. Genetic analysis of the microbial contents of ancient human feces from La Cueva de los Chiquitos Muertos, an archaeological site on the Rio Zape in Durango, Mexico, shows that these long-gone people carried microbial communities similar to those of present-day residents of remote rural areas.^3^

Using a new bioinformatic tool called SourceTracker, which compares the community of microorganisms in a sample to that of a known source, the researchers showed that the gut microbiomes found in the Rio Zape samples also matched well with that of Ötzi the Iceman, a mummy preserved in the permafrost of the Tyrolean Alps for roughly 5,200 years.^4^

But the samples also showed some notable differences from the microbial communities carried by most people today. For instance, the Rio Zape people had abundant bacteria in the genus *Prevotella*, which is associated with a diet rich in carbohydrates—and also common in the microbiomes of people from remote rural areas in Africa and Latin America.^3^ Modern, westernized people who eat a lot of animal fat and meat tend to have gut microbiomes dominated instead by *Bacterioides*.^1^^,^^5^

“Life in urban environments, with antibiotics and advanced sanitation, represents a fundamental change in our relationship with microbes,” says Cecil Lewis, an anthropologist at the University of Oklahoma and coauthor of the new study. “For the most part, we’ve benefited from that change, but it appears we’re also increasing our risk for allergies and other inflammatory diseases. The best way to understand this situation is by studying the ancestral state of the human microbiome.”

“It’s mind-blowing that the signature of ancient microbiomes can be preserved in the archeological record for thousands of years,” says Brian Kemp, a molecular anthropologist at Washington State University. The new findings, he says, offer a window into the biology of the distant past, into now-rare but perhaps still viable relationships between humans and microbes.

**Figure:**
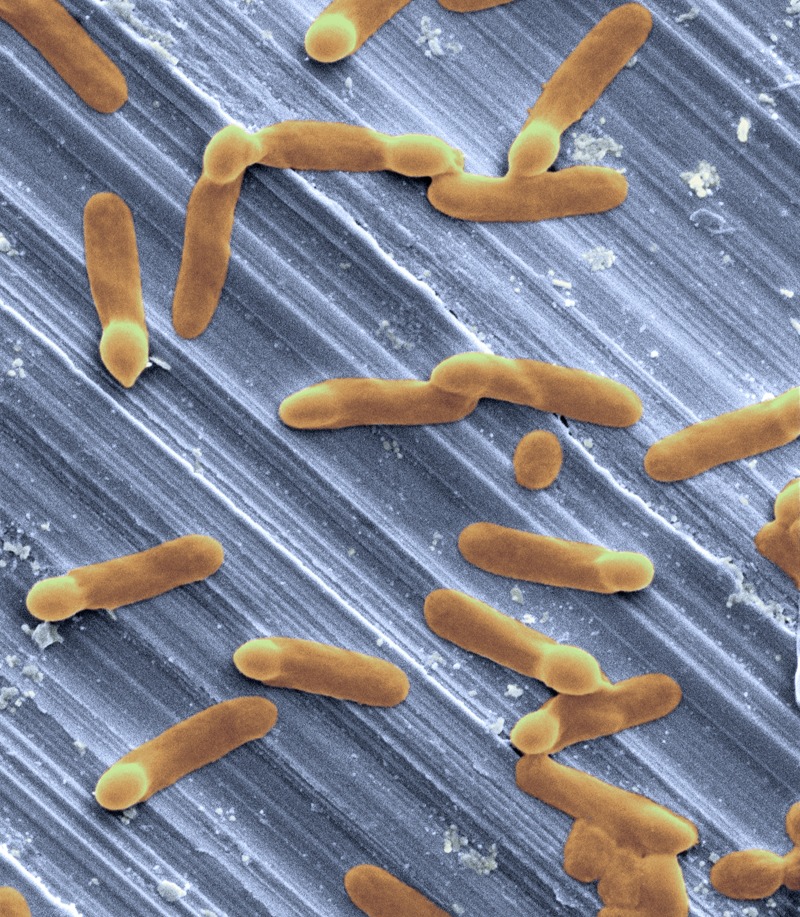
*C. difficile* on stainless steel © Jim Reed / Corbis

One example is the spirochete bacterium *Treponema*, carried by the ancients of Rio Zape and by people living today in remote communities of Africa and Latin America. Some researchers hypothesize that *Treponema* may help rural people digest their high-cellulose diet and protect them from inflammatory diseases of the colon that are common among modern urban populations.^1^ “This species has disappeared from the microbiomes of modern urbanites,” notes Lewis, but seems to be “a common microbe for people with more traditional lifestyles.”

Some recent studies, including a pioneering look at microbes preserved in dental plaque on ancient human teeth,^6^ suggest that human gut and oral microbiomes have become less stable and diverse since the domestication of plants and animals and especially since the Industrial Revolution, setting the stage for a rise in inflammatory diseases. “Humans are superorganisms—the bacterial cells in our bodies outnumber the human cells by ten to one,” Lewis says. “It’s no surprise that [microbial shifts caused by] farming, antibiotics, and industrialization have had some cost to our health.”

“Understanding these ancient microbiomes may provide some insight into how our physiology and pathophysiology has changed,” says Indi Trehan, a pediatrician at Washington University and coauthor of recent research on gut microbiomes in remote versus developed societies.^2^ “These are still early days in understanding this field—and even earlier still in applying the information provided by studies of ancient humans’ microbiomes—but I am … optimistic that we have much to learn from these studies that may be useful for our present-day problems.”

One potential example of how studies of ancient human microbiomes may eventually aid the treatment of modern diseases involves *Clostridium difficile*. This toxin-producing bacterium affects some patients who have been treated with broad-spectrum antibiotics, depleting their normal intestinal flora. A steady increase in *C. difficile* cases and evidence of antibiotic resistance make this illness a cause for concern.^7^ Infusion of feces from healthy donors has been shown to restore microbial diversity and cure *C. difficile* infection much more successfully than conventional treatment with antibiotics.^8^ Says Lewis, “Maybe down the road the study of ancient microbiomes will contribute to treating *C. difficile* and other modern illnesses in a better, more informed way.”
